# TNF-α/IL-10 Ratio Correlates with Burn Severity and May Serve as a Risk Predictor of Increased Susceptibility to Infections

**DOI:** 10.3389/fpubh.2016.00216

**Published:** 2016-10-05

**Authors:** Amy Tsurumi, Yok-Ai Que, Colleen M. Ryan, Ronald G. Tompkins, Laurence G. Rahme

**Affiliations:** ^1^Department of Surgery, Massachusetts General Hospital, Boston, MA, USA; ^2^Department of Microbiology and Immunology, Harvard Medical School, Boston, MA, USA; ^3^Shriners Hospitals for Children-Boston^®^, Boston, MA, USA; ^4^Department of Intensive Care Medicine, Inselspital, Bern University Hospital, University of Bern, Bern, Switzerland

**Keywords:** burns, injuries, biomarkers, infection, cytokines, burn severity, immune response, burn trauma

## Abstract

Severe burn injury renders patients susceptible to multiple infection episodes; however, identifying specific patient groups at high risk remains challenging. Burn-induced inflammatory response dramatically modifies the levels of various cytokines. Whether these changes could predict susceptibility to infections remains unknown. The aim of this study was to determine the early changes in the pro- to anti-inflammatory cytokine ratio and investigate its ability to predict susceptibility to repeated infections after severe burn trauma. The patient population consisted of 34 adult patients having early (≤48 h since injury) blood draws following severe (≥20% total burn surface area (TBSA)) burn injury and suffering from a first infection episode at least 1 day after blood collection. Plasma TNF-α and IL-10 levels were measured to explore the association between the TNF-α/IL-10 ratio, hypersusceptibility to infections, burn size (TBSA), and common severity scores (Acute Physiology and Chronic Health Evaluation II (APACHEII), Baux, modified Baux (R-Baux), Ryan Score, and Abbreviated Burn Severity Index (ABSI)). TNF-α/IL10 plasma ratio measured shortly after burn trauma was inversely correlated with burn size and the injury severity scores investigated, and was predictive of repeated infections (≥3 infection episodes) outcome (AUROC [95%CI] of 0.80 [0.63–0.93]). Early measures of circulating TNF-α/IL10 ratio may be a previously unidentified biomarker associated with burn injury severity and predictive of the risk of hypersusceptibility to repeated infections.

## Introduction

Burn injury, one of the major causes of trauma-related mortality worldwide, is a significant global public health concern (1). While the majority of burn patients no longer die directly from the shock induced by the burn trauma as a result of advances in modern resuscitation measures, infections and infection-related complications developed days after admission continue to pose significant risks to patients’ lives (2). Considering this negative impact of burn-related infections on mortality, patient recovery, and health-care costs, it is crucial to develop novel methods to detect and manage specific patients with increased risk of infection (3). Furthermore, the pervasiveness of indiscreet antimicrobial use in the clinics contributes to toxicity in patients and the rapid emergence of multidrug-resistant strains (4). Improved methods to identify patients most at risk of repeated infection episodes at triage have the potential to facilitate personalization of prophylactic anti-infection therapies and enhance antibiotic stewardship (5).

Severe burn trauma contributes to the pathogenesis of infections and infection-related morbidity by various direct dermal and non-dermal factors (6). There is ample evidence demonstrating that trauma-related factors of burn size [percent of total burn surface area (TBSA)] and smoke inhalation injury result in increased risk of death (7–10) and that the elimination of the dermal barrier renders extensively burned patients susceptible to bacterial, fungal, and viral infections (11). Burn extent was also previously found to correlate with increased gut permeability, which may be a non-dermal factor leading to altered immune response and infection-related outcomes (12). Severity scores have been developed for the early assessment of the risk of mortality for burn patients, including the Ryan Score (7), Abbreviated Burn Severity Index (ABSI) (13), Baux Score (14), and modified Baux (R-Baux) Score (9). The Acute Physiology and Chronic Health Evaluation (APACHEII) score is also a measure of injury severity that is generally applied to all adult Intensive Care Unit (ICU) patients (15). Previous studies have evaluated the use of these various clinical severity scores in assessing risk of infection (16–18). These scores require multiple clinical measurements and are not applied specifically for infection prediction. Therefore, it is of interest to develop early biomarkers that may further improve the identification of the subset of patients at risk of infection and related outcomes among a population of extensively burned patients, who are especially susceptible.

The effects of burn trauma at the molecular level and biomarker development have only been explored in limited depth. Recently, we identified a leukocyte transcriptome biomarker panel that could predict hypersusceptibility to infections in burn patients (19). Hypersusceptible patients showed early alterations in immune-related signaling pathways, epigenetic modulation, and chromatin remodeling. Considering the pivotal role of the immune response following burn trauma, the ability to predict adverse outcomes based on an early blood biomarker is expected to contribute significantly to the implementation of personalized management of burn patients and the use of risk stratification (3).

It is well established that major burn injuries induce acute systemic and localized activation of inflammatory pathways, leading to the release of various cytokines (20–23). These studies suggested that proinflammatory biomarkers, including tumor necrosis factor-α (TNF-α), C-reactive protein (CPR), and interleukin-6 (IL-6), may be useful for monitoring post-burn inflammation and/or immune dysfunction. A previous study found IL-4, IL-8, granulocyte macrophage colony-stimulating factor (GM-CSF), and monocyte chemotactic protein 1 (MCP-1) to be predictive of death from sepsis and multiple organ failure (MOF) (22). Another study among burn patients found an association of IL-8 levels with sepsis, MOF, and increased length of stay (24). Moreover, severely burned non-survivor children with inhalation injury displayed increased IL-4, IL-7, IL-10, and IL-13 levels at admission compared to survivors (25). IL-1RA, IL-6, and MCP-1 were also found to be predictive of mortality following severe burns (26). Although these studies found associations of altered cytokine levels with mortality and morbidity after burn trauma, no previous studies have investigated whether circulating blood cytokine biomarkers soon after injury could identify specific patients who are hypersusceptible to increased infection episodes during the course of recovery.

We specifically investigated the role of early plasma TNF-α and IL-10 as predictors of hypersusceptibility to infections after burn. TNF-α is a major proinflammatory cytokine indispensable for microbial infection control, and IL-10 is a crucial anti-inflammatory cytokine that is additionally known to negatively regulate TNF-α signaling (27, 28). Thus, it is conceivable that the balance or ratio between these cytokines may serve as a surrogate measure of pro- to anti-inflammatory immune homeostasis. Moreover, we hypothesized that assessing the ratio of TNF-α and IL-10 would aid in overcoming the challenges of inter- and intra-individual variability in leukocyte number, which is expected to make normalization of secreted plasma cytokine levels difficult (29).

The TNF-α/IL-10 ratio has been previously used as a biomarker in a few cohort studies. In a study conducted in Russia among healthy children and children with cystic fibrosis, live rubella vaccine challenge attenuated TNF-α/IL-10 ratio (30). Another study among North Indian adults found it to be elevated in the plasma of acute myocardial infarction patients compared to that of matched healthy control (31). An additional study conducted in Brazil found TNF-α/IL-10 ratio to be correlated with hyperglycemia in the plasma and placenta of pregnant women (32). While these studies demonstrate the use of the TNF-α/IL-10 ratio as a biomarker, it has yet to be assessed in the context of trauma and infection prediction.

This study aims to evaluate whether the TNF-α/IL-10 ratio, a cytokine marker of the balance between key pro- to anti-inflammatory levels, would predict susceptibility to multiple infections. Predicting the risk before the occurrence of infection has the potential to maximize the valuable window of time for early interventions for preventing or minimizing infections. The use of precision medicine that is linked to the use of genomic and transcriptome data will permit the early triage of patients and early assessment of their risk to multiple infections. Thus, it would enable the integration of predictive analytics into high-value care, as optimized treatment courses and personalized precision therapy will be delivered, in addition to antibiotic stewardship and care cost reduction.

## Materials and Methods

### Study Design/Patient and Outcome Definition

This study was performed by means of the secondary use of 573 patient clinical data from the Inflammation and the Host Response to Injury Study (“Glue Grant”), a prospective, longitudinal study which enrolled burn patients with minimum 20% TBSA at six US institutions between 2003 and 2009. Permission for this secondary use of the de-identified data was obtained from the Massachusetts General Hospital Institutional Review Board.

Among the 573 patients in the database we used, 81 had blood samples with plasma proteomic information available. From these 81 patients, a total of 34 adult (age ≥16 years) patients were analyzed based on our inclusion criteria of having not sustained electrical burn injury, with early (≤48 h since injury) blood sample collection, had first infection found not sooner than 24 h after blood draw, spent at least 1 day in the ICU, and had clinical data available. Where patients had more than one blood sample collection, the earliest collection was used for the analysis. Forty-six individuals were excluded for one or more of the following reasons: 31 were pediatric patients aged ≤16 years; 1 patient suffered from electrical burns; thus, TBSA values were considered to be not representative of the severity of their burn injury; 31 had first blood sample >48 h; 36 had first recorded infection prior to, or less than a day after first blood collection; and thus, it was considered that cytokine expression may not be predictive of but potentially a consequence of infection (Figures S1 and S2 in Supplementary Material).

The definitions of clinical outcomes are based on the guidelines outlined by the GLUE Grant Consortium that participating institutions followed (Materials and Methods in Supplementary Material). Total infection episode counts from the time of the injury were tabulated similarly to the method described in a previous publication, based on a decision tree that takes into consideration the timing of infections, type of infection, and type of pathogen isolated (19). Briefly, in order to determine whether or not a subsequent episode was independent (i.e., not an extension of a previously recorded infection), each record was considered to be on a “waiting list” for 6 days if the types and modes of infection and pathogen were similar or 2 days if they were not. In this study, the case hypersusceptible group was defined as those having experienced repeated infections (≥3 cumulative infection episodes). According to this definition of hypersusceptibility, half of the patients (14 patients) were classified as the case group.

Blood sample collection and processing methods for cytokine levels of the Glue Grant cohort was previously described (21, 22, 24). In short, peripheral blood was collected before anesthesia induction in preparation of operational intervention and processed within 1 h of blood draw by centrifugation at 400 × *g* at 22°C for 10 min for plasma isolation. These samples were stored at −80°C at the Inflammation and the Host Response to Injury Sample Collection and Coordination Site at the University of Florida College of Medicine, Gainesville, FL, USA. Plasma cytokine concentrations were quantified by the Linco Research multiplex bead array and MiraiBio software for analysis (24). For this study, the TNF-α/IL-10 ratio was calculated from picograms per milliliter of cytokine levels recorded for TNF-α and IL-10 for each patient.

The APACHEII Score (15) was recorded at the respective participating institutions. For this study, the Ryan Score (7) was assigned as previously described, where patients received a Ryan Score of 1 for each of the following risk factors – TBSA percent above 40, age above 60, and the presence of inhalation injury. ABSI was calculated by adding 1 point for male and 0 for female, adding 1 point for the presence of inhalation injury and 0 without, adding 1 point for the presence of third-degree burns and 0 without, and adding allocated points for each ABSI age category (1 point for 0–20 years, 2 points for 21–40 years, and so forth at 20-year intervals) and TBSA categories (1 point for 1–10%, 2 points for 11–20%, and so forth at 10% intervals) (13). The classic Baux Score was determined by summing the TBSA percent (14) and age. The Modified Baux (R-Baux) Score was calculated by adding the TBSA and age for patients without inhalation injury presence or an additional 17 for those with inhalation injury (9).

### Statistical Analysis

Baseline characteristics for the overall study population and for those stratified into hypersusceptible case (≥3 infection episode) and non-case (≤2 infection episode) groups are reported as means with SDs, medians with interquartile range, or proportions and percentages, as indicated in the legend. For comparing means between two groups, the two-sample equal variance *t*-test with two-tailed *p*-values was used, and for comparing means across more than one group, the one-way analysis of variance (ANOVA) was used, with Dunnett’s *post hoc* test. Medians between two groups were compared by using the Mann–Whitney test with two-tailed *p*-value. For comparing proportions, Fisher’s exact test with two-tailed *p*-values was used to account for the small sample size.

Logistic regression models were fitted by Firth’s penalized maximum likelihood method using the “logistf” package in R (33) to account for the small sample size of our study (34) for evaluating the association of the continuous predictors of TNF-α/IL-10, TBSA, APACHEII Score, Baux Score, and R-Baux, with the outcome of susceptibility to infection events (≥3 cumulative infection episodes). The 95% confidence intervals for the total area under receiver operating characteristic curve (AUROC) was constructed by stratified bootstrap with 10,000 replicates with replacement using the “pROC” package in R (35). Sensitivity and specificity 95% confidence intervals were calculated according to the Wilson score method with continuity correction (36).

### Software Used

SAS 9.3 (SAS Institute Inc., NC, USA) and R3.1.3 were used for the analyses described above.

## Results

### Patient Demographics and Baseline Characteristics

The characteristics of the 34 adult subjects included in the study are presented in Table 1 and Figure S1 in Supplementary Material. The overall study population consisted of approximately two-third males (64.7%), with an average ±SD age of 40.6 ± 17.2 years (Table 1). Our study is comprised of extensively burned patients, with patients suffering from a mean TBSA of 41.6 ± 18.8% with the great majority presenting with third-degree burns (91.2%). Forty-four percent had sustained inhalation injury, and the majority of burn injury was due to flame (67.7%). A high proportion of the patients developed infections, with the median [IQR] number of infection episodes of 3 [1–6], with 52.9% of patients having had recorded burn wound site infections and 82.4% with various types of nosocomial infections. The prevalence of the different subtypes of nosocomial infections recorded included 55.9% with pneumonia, 38.2% bloodstream infections, 44.1% with urinary tract infection, 2.9% endocarditis, 5.9% pseudomembranous colitis, and 17.6% catheter-related bloodstream infections. Various clinical severity scores were calculated. Overall, patients had mean general APACHEII Severity Score of 19.0 ± 8.2 and burn-specific severity scores of 82.1 ± 23.9 Baux Score and 89.6 ± 26.4 R-Baux Score (Table 1). For the categorical severity scores, the patients had median [IQR] Ryan Score of 1 [0–2] and ABSI of 9 [7–11]. Patients arrived at the hospital soon after injury, with average arrival time of 4.1 ± 2.5 h since injury and average blood collection time of 19.9 ± 12.0 h since injury. The average TNF-α/IL-10 cytokine ratio was 0.134 ± 0.136, and average time to first recorded infection was 5.4 ± 3.0 days post-burn. The risk of death overall was 17.6%, and the average length of hospital stay was 55.0 ± 53.4 days among survivors.

**Table 1:** **Baseline characteristics, where results are shown**.

	All patients (*N* = 34)	Non-cases: ≤2 infection episode (*N* = 17)	MIE CASES: ≥3 infection episodes (*N* = 17)	*p*-value
(1) Demographics				
Age (years)^a^	40.6 ± 17.2	38.4 ± 16.5	42.8 ± 18.2	0.464
Sex (male)^c^	22 (64.7%)	12 (70.6%)	10 (58.9%)	0.721
BMI, continuous (kg/m^2^)^a^	26.3 ± 6.9	26.7 ± 6.0	25.9 ± 7.9	0.765
Time since injury before admission (h)^a^	4.1 ± 2.5	3.9 ± 2.7	4.4 ± 2.3	0.513
(2) Characteristics/severity of burn injury^c^				0.364
Etiology: flame	23 (67.7%)	10 (58.8%)	13 (76.5%)	0.465
Flash	5 (14.7%)	2 (11.8%)	3 (17.7%)	1.000
Scald	2 (5.9%)	2 (11.8%)	0 (0%)	0.485
Other	4 (11.8%)	3 (17.7%)	1 (5.9%)	0.601
TBSA (%)^a^	41.6 ± 18.8	31.9 ± 11.2	51.2 ± 20.0	0.0015
Second-degree burn (yes)^c^	27 (79.4%)	13 (76.5%)	14 (82.4%)	1.000
Third-degree burn (yes)^c^	31 (91.2%)	14 (82.4%)	17 (100%)	0.227
Inhalation injury (yes)^c^	15 (44.1%)	5 (29.4%)	10 (58.8%)	0.166
APACHEII^a^	19.0 ± 8.2	14.1 ± 7.8	23.8 ± 5.2	0.0003
Baux^a^	82.1 ± 23.9	70.3 ± 13.7	94.0 ± 26.2	0.0002
R-Baux^a^	89.6 ± 26.4	75.3 ± 16.1	104.0 ± 27.1	0.0007
Ryan Score^b^	1 [0–2]	1 [0–1]	1 [1–2]	0.0022
Abbreviated Burn Severity Index (ABSI)^b^	9 [7–11]	7 [7–9]	11 [9–11]	0.0005
(3) Plasma collection				
First blood collection since injury (h)^a^	19.9 ± 12.0	20.4 ± 12.0	19.3 ± 12.4	0.796
TNF-α/IL-10 ratio^a^	0.134 ± 0.136	0.200 ± 0.154	0.067 ± 0.072	0.0029
(4) Infections-related outcomes				
First infection day since injury^d^ (days)^a^	5.4 ± 3.0	5.8 ± 3.6	5.2 ± 2.7	0.573
Number of infection events^b^	3 [1–6]	1 [0–2]	6 [5–10]	<0.0001
Burn wound infection (yes)^c^	18 (52.9%)	3 (17.6%)	15 (88.2%)	<0.0001
Nosocomial infection^e^ (yes)^c^	28 (82.4%)	12 (70.6%)	16 (94.1%)	0.175
Pneumonia (yes)^c^	19 (55.9%)	5 (29.4%)	14 (82.4%)	<0.0001
Bloodstream infection (yes)^c^	13 (38.2%)	1 (5.9%)	12 (85.7%)	0.0002
Urinary tract infection (yes)^c^	15 (44.1%)	5 (29.4%)	10 (58.8%)	0.166
Endocarditis (yes)^c^	1 (2.9%)	0 (0%)	1 (100%)	1.000
Pseudomembranous colitis (yes)^c^	2 (5.9%)	0 (0%)	2 (100%)	0.485
Catheter-related bloodstream infection (yes)^c^	6 (17.6%)	1 (5.9%)	5 (29.4%)	0.175
Other (yes)^c^	7 (20.6%)	2 (11.8%)	5 (29.4%)	0.398
(5) Outcomes				
Death (yes)^c^	6 (17.6%)	1 (5.9%)	5 (29.4%)	0.175
Hospital stay length^f^ (days)^a^	55.0 ± 53.4	27.2 ± 15.4	92.2 ± 63.7	0.0005

*^a^Mean ± SD with t-test two-tailed p-values*.

*^b^Median [IQR] with Mann–Whitney two-tailed *p*-values*.

*^c^Number of patients (%) with Fisher’s exact two-tailed *p*-values*.

*^d^Among those with at least one recorded infection*.

*^e^Nosocomial infections exclude burn wound infections and include the subcategory recording described below it*.

*^f^Among survivors (*n* = 28)*.

Demographic characteristics, including age, sex, BMI, mechanisms of injury, were not significantly different between hypersusceptible and control patients (Table 1). On the other hand, hypersusceptible patients had significantly higher measures of injury severity, including TBSA (mean 31.9 ± 11.2% for non-cases versus 51.2 ± 20.0% for cases, *p* = 0.0015), APACHEII score (mean 14.1 ± 7.8 for non-cases versus 23.8 ± 5.2 for cases, *p* = 0.0003), Baux (mean 70.3 ± 13.7 for non-cases versus 94.0 ± 26.2 for cases, *p* = 0.0002), R-Baux (mean 75.3 ± 16.1 for non-cases versus 104.0 ± 27.1 for cases, *p* = 0.0007), Ryan Score (median 1 [1–0] for non-cases versus 1 [1–2] for cases, *p* = 0.0022), and ABSI (median 7 [7–9] for non-cases versus 11 [9–11] for cases, *p* = 0.0005). Although there was slightly increased proportion of patients having suffered third-degree burns among the case group, it is not significant (82.4% for non-cases versus 100% for cases, *p* = 0.227), presumably due to the high overall prevalence among our particular severely injured patient group. The prevalence of inhalation injury was higher in the hypersusceptible patients, although not significant (29.4% for non-cases versus 58.8% of cases, *p* = 0.166).

The biomarker of interest, plasma cytokine TNF-α/IL-10 ratio, was significantly lower in the hypersusceptible patient group (mean 0.200 ± 0.154 for non-cases versus 0.067 ± 0.072 for cases, *p* = 0.0029), despite comparable hospital admission time (mean 3.9 ± 2.7 h since injury for non-cases versus 4.4 ± 2.3 h since injury for cases, *p* = 0.513) and first blood collection time between the two groups (mean 20.4 ± 12.0 h since injury for non-cases versus 19.3 ± 12.4 h since injury for cases, *p* = 0.796). The first recorded infection times were also similar between the hypersusceptible and case groups (mean 5.8 ± 3.6 days since injury for non-cases versus 5.2 ± 2.7 for cases, *p* = 0.573). Among survivors, days of stay in the hospital were significantly longer for patients with repeated infections (mean 27.2 ± 15.4 for non-cases versus 92.2 ± 63.7 for cases, *p* = 0.0005), as expected.

### Burn Injury Severity Is Inversely Correlated with TNF-α/IL-10 Cytokine Ratio

**Figure 1:**
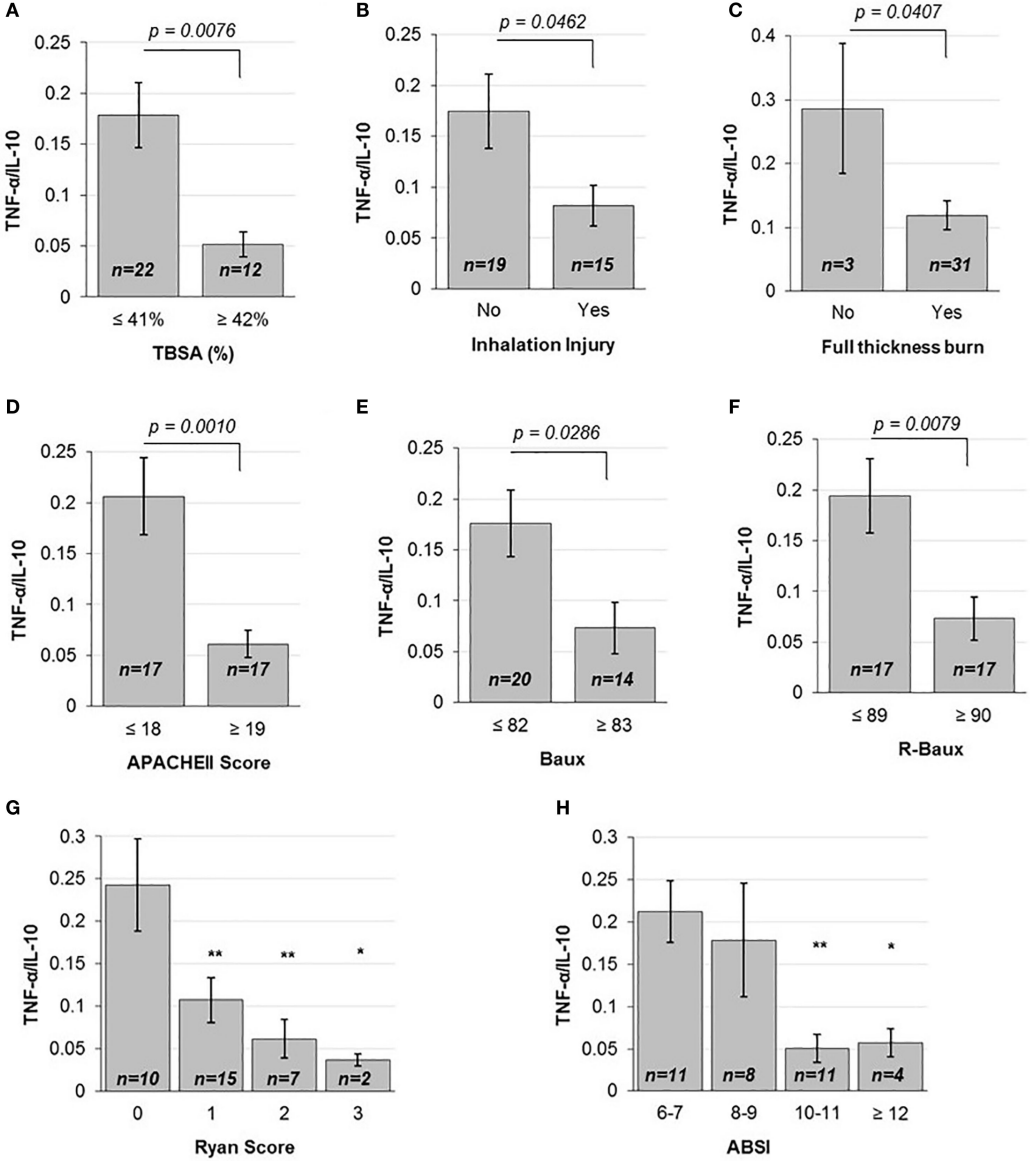
**Various injury severity measures are inversely correlated to TNF-α/IL10**. **(A)** TBSA percent above the mean, **(B)** the presence of inhalation injury and **(C)** full-thickness burn, above mean **(D)** APACHEII Score, **(E)** Baux and **(F)** R-Baux, and each increasing **(G)** Ryan Score and **(H)** ABSI categories show significantly decreased TNF-α pro- to IL-10 anti-inflammatory plasma cytokine ratio for most (SE as error bars, *t*-test two-sided *p*-values indicated, or one-way ANOVA with Dunnett’s *post hoc* test, *p < 0.1 and **p < 0.05 compared to the first category).

In order to determine the correlation between the severities of burn injury to the levels of early pro- versus anti-inflammatory state as ascertained by the early plasma TNF-α/IL-10 ratio, we first stratified patients according to mean TBSA, inhalation injury, and full-thickness burn status. Patients below the mean TBSA (≤41%) had average ±SD early TNF-α/IL-10 ratio level of 0.178 ± 0.150, compared to more extensively burned patients with above mean TBSA (≥42%) that had 0.052 ± 0.042 (*p* = 0.0076) (Figure 1A). Stratifying patients into categories according to the first quartile, median, and third quartile TBSA also showed a progressive decrease in TNF-α/IL-10 ratio, from 0.206 ± 0.142 to 0.179 ± 0.193 to 0.1012 ± 0.07 to 0.0439 ± 0.04 (*p* < 0.05 from below the first quartile to above the third quartile) (Figure S3A in Supplementary Material). Those who did not suffer inhalation injury had mean 0.175 ± 0.159 TNF-α/IL-10 cytokine ratio, compared to those who did, who had mean TNF-α/IL-10 cytokine ratio of 0.082 ± 0.078 (*p* = 0.0462) (Figure 1B). The majority of patients in our dataset had full-thickness burn; however, compared to those without, whose mean TNF-α/IL-10 cytokine ratio was 0.286 ± 0.177, those who had suffered full-thickness burns had mean TNF-α/IL-10 cytokine ratio of 0.119 ± 0.126 (*p* = 0.0407) (Figure 1C). Together, these data indicate that individual measures of burn injury correlates with early TNF-α/IL-10 cytokine ratio.

We then compared patients according to the continuous injury severity scores of the general APACHEII score and burn-specific Baux and R-Baux scores. Patients who had APACHEII score below the mean (≤18) had mean TNF-α/IL-10 cytokine ratio of 0.206 ± 0.156, compared to patients with above mean (≥19) APACHEII who had mean TNF-α/IL-10 cytokine ratio of 0.061 ± 0.055 (*p* = 0.001) (Figure 1D). When stratifying patients according to quartile categories of APACHEII, the TNF-α/IL-10 cytokine ratio decreased progressively, from 0.238 ± 0.176 to 0.171 ± 0.132 to 0.070 ± 0.055 to 0.049 ± 0.055 (*p* < 0.05 from below the first quartile to between the median and third quartile, and *p* < 0.01 from below the first quartile to above the third quartile) (Figure S3B in Supplementary Material). The group of patients with below mean Baux Score (≤82) had mean TNF-α/IL-10 cytokine ratio of 0.176 ± 0.147 compared to patients with above mean (≥83) Baux of 0.0734 ± 0.094 (*p* = 0.0286) (Figure 1E). Patients with below mean R-Baux Score (≤89) had mean TNF-α/IL-10 cytokine ratio of 0.194 ± 0.152 compared to patients with above mean (≥90) R-Baux of 0.0735 ± 0.088 (*p* = 0.0079) (Figure 1F).

Additionally, we evaluated patients across the categorical injury severity measures of the Ryan Score and ABSI. Our patient group’s Ryan Score varied from the lowest 0 to the maximum of 3. Each increase in Ryan Score resulted in progressive decrease in TNF-α/IL-10 cytokine ratio compared to the basal score of 0, of mean TNF-α/IL-10 cytokine ratio of 0.243 ± 0.171 to Ryan Score 1 with mean TNF-α/IL-10 cytokine ratio of 0.107 ± 0.102 to Ryan Score 2 with mean TNF-α/IL-10 cytokine ratio of 0.060 ± 0.023 to Ryan Score 3 with mean TNF-α/IL-10 cytokine ratio of 0.037 ± 0.010 (*p* < 0.05 from 0 to 1, and from 0 to 2, and *p* < 0.1 from 0 to 3) (Figure 1G). For the ABSI score, the lowest corresponded to the category of “moderately severe” (ABSI score 6–7), to “serious” (ABSI score 8–9), “severe” (ABSI score 10–11), and “maximum” (ABSI score ≥12) severity. Categorizing patients according to increased ABSI severity category found decreased levels of mean TNF-α/IL-10 cytokine ratio from the “moderately severe” category of 0.212 ± 0.121 to the “serious” category of 0.179 ± 0.189 to the “severe” category of 0.0508 ± 0.055, and the “maximum” severity category of 0.057 ± 0.033 (*p* < 0.05 from “moderately severe” to “serious” and *p* < 0.1 from “moderately severe” to “maximum”) (Figure 1H). Collectively, these data demonstrate overall that mean TNF-α/IL-10 cytokine ratio is inversely correlated with the various burn injury severity measures described above and may reflect a state of immunosuppression.

### Early Plasma TNF-α/IL-10 Cytokine Ratio Maybe a Novel Biomarker to Predict Increased Risk to Multiple Infection Episodes

**Figure 2:**
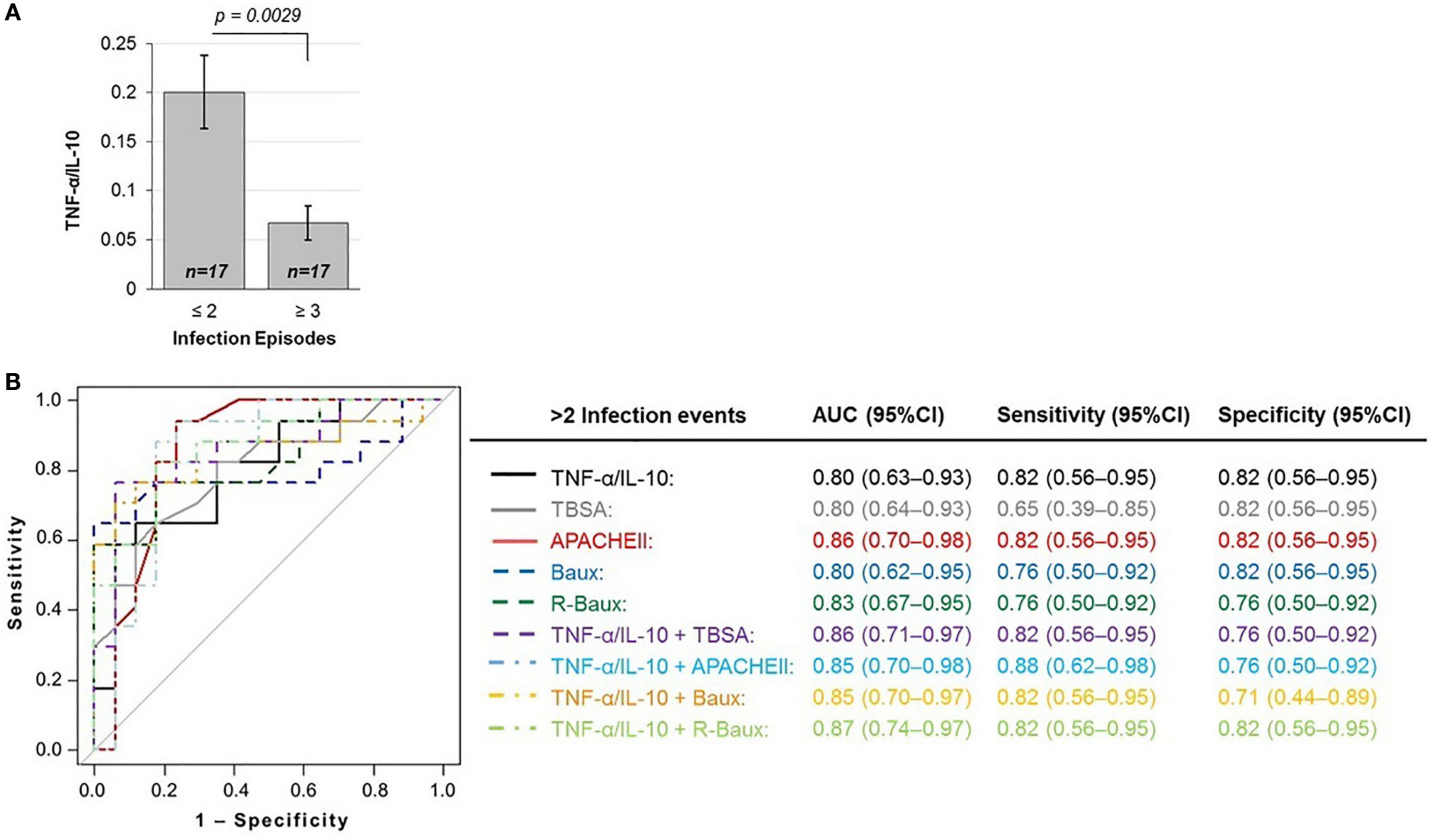
**TNF-α/IL-10 protein ratio is inversely correlated with (A) susceptibility to infections and predictive of the outcome of repeated infections (≥3 infection episodes), as shown by the (B) total area under the ROC curve, sensitivity, and specificity of various logistic regression models**.

The baseline measure of TNF-α/IL-10 cytokine ratio of patients who were less susceptible to multiple infection episodes during the course of their recovery (≤2 total episodes) was 0.200 ± 0.154, significantly higher compared to hypersusceptible patients who experienced repeated infection episodes (≥3 total episodes), with mean TNF-α/IL-10 cytokine of 0.067 ± 0.072 (*p* = 0.0029) (Figure 2A). In order to assess whether or not early circulating plasma TNF-α/IL-10 ratio may indeed be a novel predictive biomarker for hypersusceptibility to infections, we assessed the AUROC of the various logistic models with TNF-α/IL-10, TBSA, and the various continuous severity scores (Figure 2B). Using TNF-α/IL-10 ratio as the sole predictor yielded an AUROC (95% CI) of 0.80 (0.63–0.93), similarly to TBSA of 0.80 (0.64–0.93), APACHEII of 0.86 (0.70–0.98), Baux of 0.80 (0.62–0.95), and R-Baux of 0.83 (0.67–0.95). Combining the various clinical with the TNF-α/IL-10 ratio resulted in AUROCs (95% CI) for TNF-α/IL-10 + TBSA of 0.86 (0.71–0.97), TNF-α/IL-10 + APACHEII of 0.85 (0.70–0.98), TNF-α/IL-10 + Baux of 0.85 (0.70–0.97), and TNF-α/IL-10 + R-Baux of 0.87 (0.74–0.97). The model with a single predictor of TNF-α/IL-10 and APACHEII and combined TNF-α/IL-10 + R-Baux had the highest sensitivity (95% CI) without compromising specificity (95% CI), of each having 0.82 (0.56–0.95) for both sensitivity and specificity. Although the combined model of TNF-α/IL-10 + APACHEII had the highest sensitivity of 0.88 (0.62–0.98), its specificity was decreased, with 0.76 (0.50–0.92). The TNF-α/IL-10 + Baux had equally high sensitivity of 0.82 (0.56–0.95); however, the specificity was decreased to 0.71 (0.44–0.89). The single predictors of R-Baux had sensitivity and specificity of 0.76 (0.50–0.92) and Baux had equal sensitivity, however, higher specificity of 0.82 (0.56–0.95). Although TBSA had the lowest sensitivity of 0.65 (0.39–0.85), its specificity was among the highest of the models, with 0.82 (0.56–0.95).

### Various Microbes Are Prevalent among Severely Burned Patients

**Figure 3:**
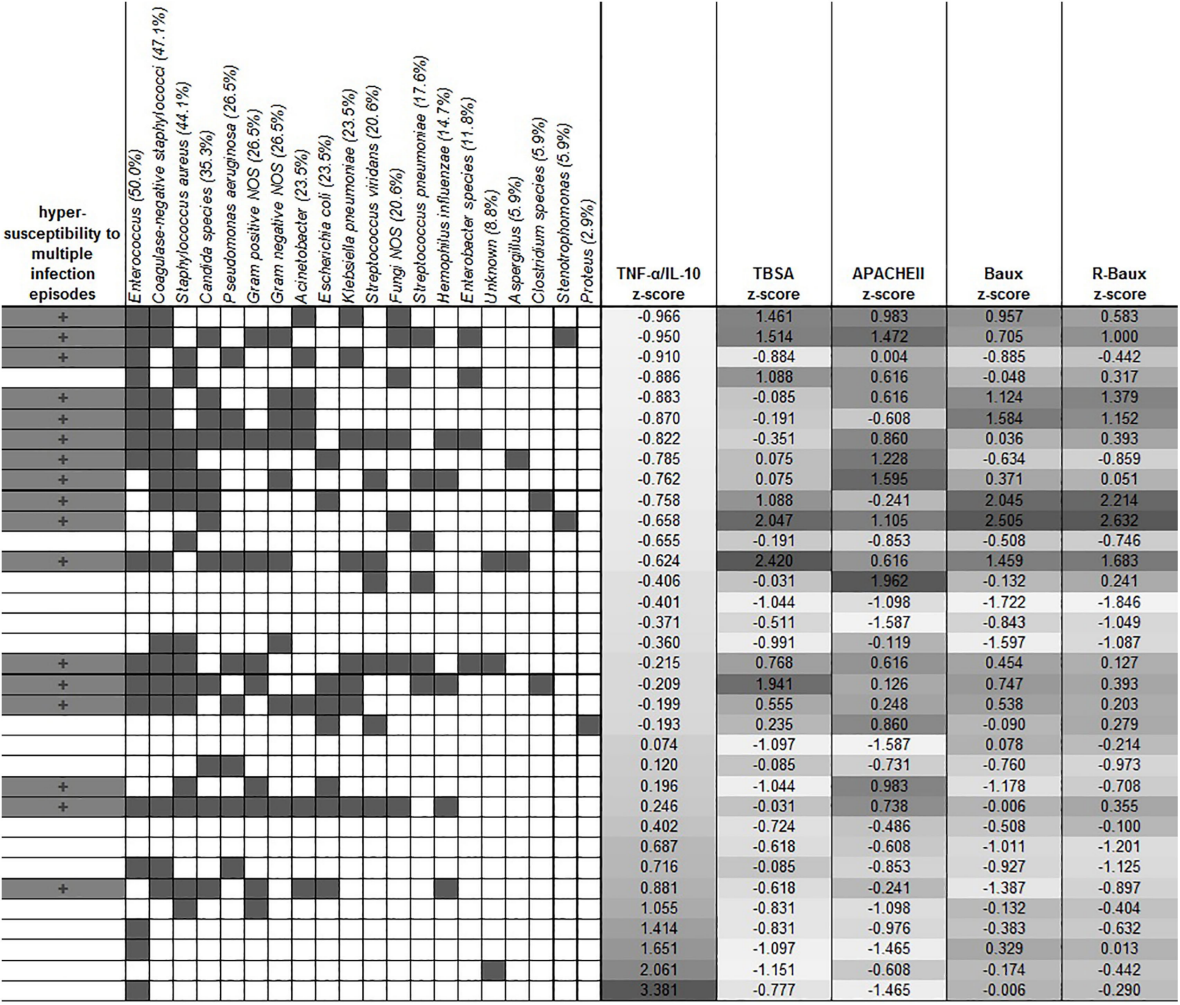
**Clustering of the different types of microbes with hypersusceptibility status**. The overall prevalence of each microbe is indicated in the parentheses in each microbe’s label. Dark gray filling indicates positive hypersusceptibility status and presence of specific microbes. TNF-α/IL-10 was sorted by the *z*-score of the population, and the corresponding severity score *z*-scores are shown in the columns as indicated.

Various microbes tested were detected, with *Enterococcus*, coagulase-negative staphylococci and *Staphylococcus aureus* being among the highest prevalence among our overall patient population (50.0%, 47.1, and 44.1 respectively). Other microbes detected in descending order of prevalence, include *Candida* species (35.3%), *Pseudomonas aeruginosa* (26.5%), Gram-positive NOS (26.5%), Gram-negative NOS (26.5%), *Acinetobacter* (23.5%), *Escherichia coli* (23.5%), *Klebsiella pneumoniae* (23.5%), *Streptococcus viridans* (20.6%), Fungi NOS (20.6%), *Streptococcus pneumoniae* (17.6%), *Hemophilus influenza* (14.7%), *Entrobacter* species (11.8%), unknown (8.8%), *Aspergillus* (5.9%), *Clostridium* species (5.9%), *Stenotrophomonas* (5.9%), and *Proteus* (2.9%) (Figure 3). In order to determine whether early TNF-α/IL-10 level is associated with the risk of a patient later developing infection to specific pathogens, we sorted the patients according to their TNF-α/IL-10 ratio *z*-scores. Clusters of pathogens appeared to group according to the hypersusceptibility status rather than the TNF-α/IL-10-fold change levels, suggesting that it is likely a risk factor for repeated infections in general, rather than to specific pathogens. In some cases, patients with relatively lower clinical severity scores had relatively higher TNF-α/IL-10 levels and *vice versa*, as determined by the population *z*-score for each of the continuous severity scores, suggesting that an individual patient’s response to injury may also play a distinct role.

## Discussion

Our study demonstrates that burn trauma severity is inversely correlated with early blood plasma pro- to anti-inflammatory signaling ratio, perhaps due to a state of immunosuppression that accompanies severe trauma. This finding is strengthened by our study design, in which we conducted our analyses using several different measures of injury severity. Although the cytokine ratio and the various severity scores were significantly inversely correlated, in some selected patients, the measured TNF-α/IL-10 ratio was high, despite high injury severity measures and *vice versa*. This observation may argue for the possibility that TNF-α/IL-10 is not merely a reflection of injury severity but also plays a distinct role in individuals’ response to injury and susceptibility to infections. Control samples of plasma from the base population prior to burn injury would enable our study to distinguish between these possibilities; however, such sample is impossible to obtain. It is also important to note that the current study base consists of a particularly critically ill patient population, who are severely (≥20% TBSA) burned with an overall high proportion having experienced inhalation injury and full-thickness burns.

It may also be informative to further investigate mechanistic aspects of cytokine regulation among burn patients, which is expected to be complex and likely mediated by various direct dermal and non-dermal processes. TBSA was previously found to correlate with increased gut permeability, which may be one mechanism leading to altered immune response by non-dermal processes, and it would be of interest to assess the relationship between cytokine biomarkers and gut permeability (12). Moreover, it has been reported that insulin attenuates cytokine response in a burn wound infection model (37), and thus, perhaps further studies of the immune response upon severe burns in diabetic patients may also be informative. Studies using animal models of burn have found that inhalation injury impacts the systematic immune response, independently of burn injury (38, 39), and in human cohort studies, increased severity of inhalation injury was also correlated with cytokine induction (40, 41). Our current study that found TNF-α/IL10 cytokine ratio to be significantly lower in patients with inhalation injury supports such notion that the localized sustained trauma often experienced by burn patients may also contribute to the systemic cytokine response. It would therefore be beneficial to further dissect the precise mechanisms, resulting in systemic immunosuppression by burn injury.

In addition to establishing that TNF-α/IL-10 ratio is inversely correlated with burn injury severity measures of TBSA, inhalation injury status, APACHEII, Baux, R-Baux, Ryan, and ABSI scores, we found that it is a biomarker that predicts increased risk of hypersusceptibility to infection during the course of recovery. Given the role of the systematic immune response in combating pathogenic encounters, our finding is mechanistically plausible. Moreover, our observations are strengthened by the use of early blood samples, prior to infections. Since the lack of fast and sensitive pathogen detection methods in the current clinical setting impose complications on epidemiological studies of infection, in our current study, we analyzed only patients whose time to first recorded infection was more than a day after the time of blood collection. Furthermore, the average time to first recorded infection among patients in our dataset was approximately 5 days since injury. Thus, given our study design, we expect that our results on the association of TNF-α/IL10 cytokine ratio are predictive of later infections, rather than a consequence of pathogenic encounter. Various pathogens were detected among patients, including species that are commonly antibiotic resistant and present a major threat to patients’ recovery process in the hospital setting, including ESKAPE pathogens, *Enterococcus, S. aureus, K. pneumoniae*, *Acinetobacter baumannii*, and *P. aeruginosa*. Our current study which analyzes the risk of patients’ susceptibility to repeated infections during recovery aims to contribute to enhanced methods of personalized medicine and antibiotic stewardship for limiting the inappropriate use of antibiotics.

Previous studies investigating cytokines following burn injury have mainly focused on each anti- or proinflammatory cytokine individually. A study among severely burned adult and pediatric populations plotted various average cytokine levels over time (21, 22). Moreover, a study found increased levels of certain cytokines at admission among patients who later died of sepsis, compared to patients who did not experience sepsis (20). Burn size categories were correlated with differential expression of several cytokines and organ function measures of cardiac output and liver size, among children (42). An additional study investigated the association of plasma IL-8 during the first 60 days, among severely burned patients in whether or not an infection was ever recorded, the occurrence of sepsis, MOF, and mortality, among pediatric patients (24). It is well established from animal and cell culture studies that anti- and proinflammatory cytokines are coregulated and interdependent and that burn trauma leads to increased differentiation and accumulation of leukocytes (43). Thus, the secreted cytokine level in plasma may differ between individuals based on innate variability of leukocyte numbers as a consequence of the severity of injury. Our current study overcomes this variability by our approach of assessing the relative pro- to anti-inflammatory cytokine ratio to allow for normalization, thereby negating observed differences between individuals, which is potentially a reflection of mere leukocyte number. Moreover, severe clinical outcomes, such as death and MOF, may be a consequence of various complications, and our study focusing on a well-defined, distinct outcome of repeated infections (≥3 infection episodes) is unparalleled.

Studies of TNF-α/IL-10 ratio in humans found that this ratio is attenuated following live rubella vaccine challenge among children (30), is elevated in the plasma of acute myocardial infarction patients compared to matched healthy control (31), and is associated with hyperglycemic states in placental tissues, with type 2 diabetes mellitus patients having the highest level (32). However, our unique approach of investigating infections by our recently defined method of quantifying cumulative infection episodes (19), prior to first recorded infection as an improved method, provides additional insight on the putative effectiveness of early circulating plasma TNF-α/IL-10 ratio as an important biomarker. In this study, TNF-α/IL-10 ratio was found to be highly predictive of hypersusceptibility to infections, with high AUROC and high sensitivity and specificity. Our results strongly suggest that TNF-α/IL-10 plasma cytokine level, given its established role, is a mechanistically feasible biomarker that warrants further studies in burn trauma and infections. Since the AUROC and/or the sensitivity of the combined model of clinical scores with TNF-α/IL-10 was increased compared to the clinical model alone, we conclude that TNF-α/IL-10 may have some effect independently of injury severity. While our study demonstrates the putative usefulness of the TNF-α/IL-10 ratio in predicting hypersusceptibility to infections in general, we did not find strong evidence for specificity toward specific pathogen types. It is also possible that differences in pathogens were not found due to the complicated nature of polymicrobial infections that patients often experience while recovering in the hospital. However, since TNF-α and IL-10 are major pro- and anti-inflammatory cytokines found to be involved in a variety of infections in animal and cell culture models, it is feasible that it plays a general role widely, rather than specifically toward select pathogens. Considering the ease of harvesting plasma and requiring merely two individual cytokine measures, as compared to the clinical scores that require multiple measurements over time, we suggest that it is important to revalidate our findings in other cohorts.

A major limitation of our current study is the relatively low number of patients who were eligible to be enrolled in the study according to our unique approach that takes into consideration the temporality of burn injury, blood collection, and first infection episode. Future revalidation studies will analyze a larger number of patients. However, despite the small size of our study, our design using multiple different measures of injury severity to assess correlations with TNF-α/IL-10 levels and the biological feasibility of our findings in relation to microbial pathogenesis gives us confidence in the relevance of our findings. It would also be of interest to conduct a study investigating TNF-α/IL-10 levels among pediatric burn patients, who may have a different immunological response. Considering our finding that APACHEII, a general severity score, was also associated with TNF-α/IL-10 similarly to TBSA and other burn-specific scores, our results may be applicable widely to other modes of severe injury. We therefore propose that early plasma TNF-α/IL-10 cytokine ratio may serve as a useful, risk predictor for infection hypersusceptibility in severe trauma in general.

## Author Contributions

AT, Y-AQ, CR, and LR conceived and designed the study. RT contributed to data collection. Y-AQ, CR, and LR oversaw the study. AT conducted the analyses, interpreted the results, and drafted the manuscript. AT, Y-AQ, CR, and LR critically revised and prepared the manuscript. AT, Y-AQ, CR, RT, and LR approved the manuscript for submission.

## Conflict of Interest Statement

The authors declare that the research was conducted in the absence of any commercial or financial relationships that could be construed as a potential conflict of interest.
