# Supplementary material for: TNF-α/IL-10 Ratio Correlates with Burn Severity and May Serve as a Risk Predictor of Increased Susceptibility to Infections
Source: Front Public Health. 2016 Oct 5;4:216. doi: 10.3389/fpubh.2016.00216 (PMC5050217; doi:10.3389/fpubh.2016.00216)
Supplement: Supplementary file 1 [file Data_Sheet_1.PDF]

*Supplementary Material*

# TNF-α/IL-10 ratio reflects burn severity and may serve as a risk predictor of increased susceptibility to infections

**Amy Tsurumi, PhD, Yok-Ai Que MD, Ph.D, Colleen M. Ryan, MD, Ronald G. Tompkins, MD, ScD, Laurence G. Rahme***

*** Correspondence:** Laurence G. Rahme: rahme@molbio.mgh.harvard.edu

## Supplementary Figures

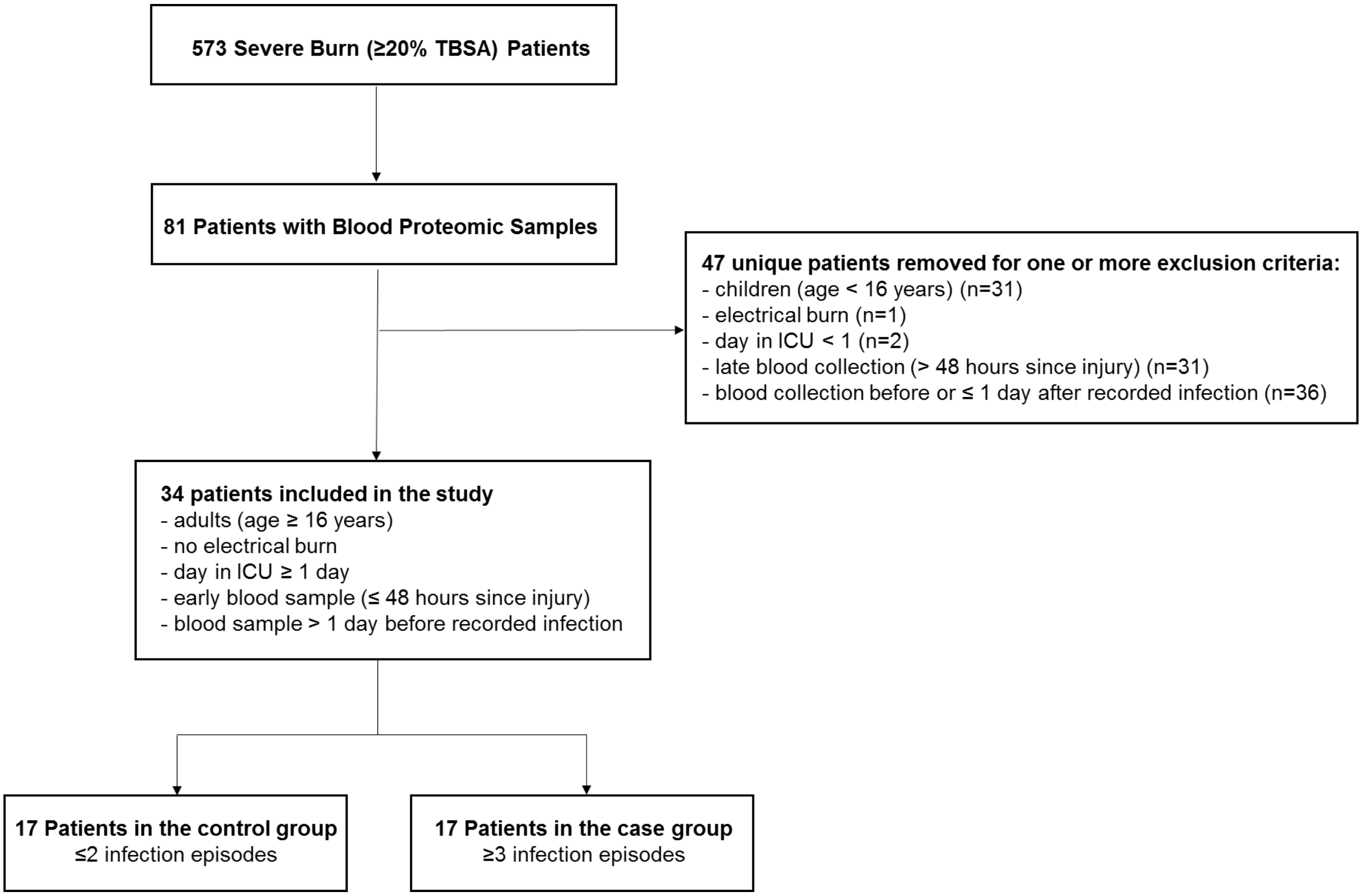


**Supplementary Figure 1.** Description of the patients included in the study.

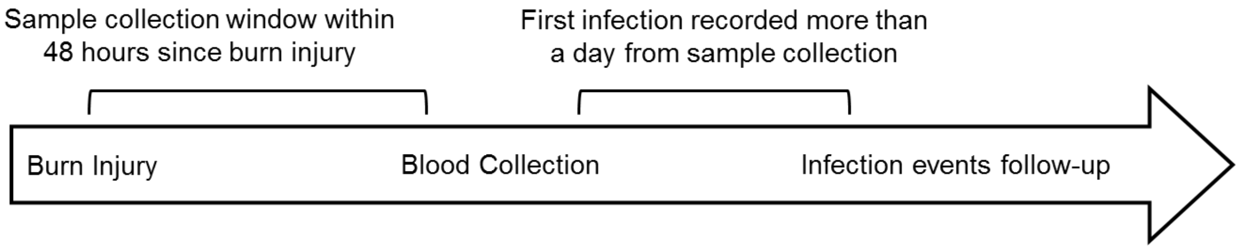


**Supplementary Figure 2.** Description of the study design.

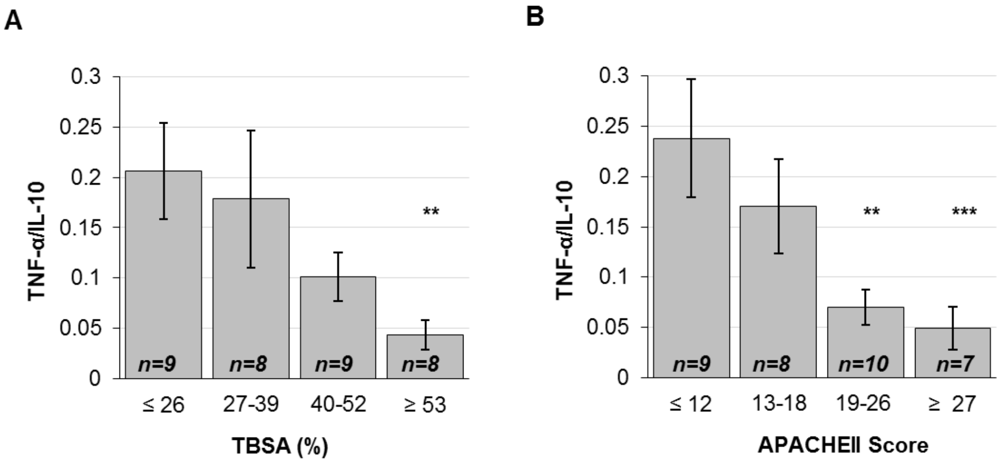


**Supplementary Figure 3.** Severity measure quartile categories of **(A)** TBSA percent and **(B)** APACHEII Score show signficantly decreased TNF-α/IL-10 plasma cytokine ratio (standard error as error bars, t-test two-sided p-value, or one-way ANOVA with Dunnett's post-hoc test. **indicates p<0.05 and ***indicates p<0.01 compared to the first category).

## Supplementary Materials and Methods

### Measurement/definition of clinical outcomes

Diagnosis of inhalation injury was determined by physical examination, followed by bronchoscopy where possible as previously described [1]. Where bronchoscopy was not performed, the following clinical criteria were considered: burns of the head and neck, burn injury occurring in a closed space vicinity, carboxyhemoglobin >15% if obtained in a timely manner after burn injury, carbonaceous sputum, and burns and/or soot in the oropharynx. Bronchoscopy was conducted in patients with these clinical criteria among intubated patients, where possible. For bronchoscopic confirmation, the

minimal criteria for diagnosis was observation of either patchy areas of erythema or carbonaceous deposits in the proximal and/or distal bronchi. APACHEII severity score [2] was calculated and recorded at the respective institutions. For assigning BMI categories (underweight, healthy, overweight, obese), patients were classified according to the Centers for Disease Control and Prevention's recommendations, where BMI less than 18.5 kg/m2 was considered underweight, 18.5 kg/m2 to 24.9 kg/m2 was considered healthy, 25.0 kg/m2 to 29.9 kg/m2 was considered overweight and 30.0 kg/m2 and above was considered obese [3].

1. Inflammation and the Host Response to Injury Investigators. Clinical Guidelines for the Diagnosis of Inhalation Injury. 2004, Sept. Retrieved Feb 1, 2015, from http://www.gluegrant.org.

2. Knaus, W.A., et al., APACHE II: a severity of disease classification system. Crit Care Med, 1985. 13(10): p. 818-29.

3. Ogden, C.L., et al., Centers for Disease Control and Prevention 2000 growth charts for the United States: improvements to the 1977 National Center for Health Statistics version. Pediatrics, 2002. 109(1): p. 45-60.
